# Target-controlled infusion of remimazolam effect-site concentration for total intravenous anesthesia in patients undergoing minimal invasive surgeries

**DOI:** 10.3389/fmed.2024.1364357

**Published:** 2024-04-17

**Authors:** Jin Young Chon, Kwon Hui Seo, Jaesang Lee, Subin Lee

**Affiliations:** Department of Anesthesiology and Pain Medicine, Yeouido St. Mary’s Hospital, College of Medicine, The Catholic University of Korea, Seoul, Republic of Korea

**Keywords:** remimazolam, anesthesia, general, sedation, pharmacodynamics

## Abstract

**Background:**

Although pharmacokinetic and pharmacodynamic models of remimazolam have been developed, their clinical application remains limited. This study aimed to administer a target-controlled infusion (TCI) of remimazolam at the effect-site concentration (Ce) in patients undergoing general anesthesia and to investigate the relationship of the remimazolam Ce with sedative effects and with recovery from general anesthesia.

**Methods:**

Fifty patients aged 20–75 years, scheduled for minimally invasive surgery under general anesthesia for less than 2 h, were enrolled. Anesthesia was induced and maintained using Schüttler’s model for effect-site TCI of remimazolam. During induction, the remimazolam Ce was increased stepwise, and sedation levels were assessed using the Modified Observer’s Assessment of Alertness/Sedation (MOAA/S) scale and bispectral index (BIS). Following attainment of MOAA/S scale 1, continuous infusion of remifentanil was commenced, and rocuronium (0.6 mg/kg) was administered for endotracheal intubation. The target Ce of remimazolam and the remifentanil infusion rate were adjusted to maintain a BIS between 40 and 70 and a heart rate within 20% of the baseline value. Approximately 5 min before surgery completion, the target Ce of remimazolam was reduced by 20–30%, and anesthetic infusion ceased at the end of surgery. Nonlinear mixed-effects modeling was employed to develop pharmacodynamic models for each sedation level as well as emergence from anesthesia.

**Results:**

The remimazolam Ces associated with 50% probability (Ce_50_) of reaching MOAA/S scale ≤4, 3, 2, and 1 were 0.302, 0.397, 0.483, and 0.654 μg/mL, respectively. The Ce_50_ values for recovery of responsiveness (ROR) and endotracheal extubation were 0.368 and 0.345 μg/mL, respectively. The prediction probabilities of Ce and BIS for detecting changes in sedation level were 0.797 and 0.756, respectively. The sedation scale significantly correlated with remimazolam Ce (*r* = −0.793, *P* < 0.0001) and BIS (*r* = 0.914, *P* < 0.0001). Age significantly correlated with Ce at MOAA/S1 and ROR.

**Conclusion:**

Effect-site TCI of remimazolam was successfully performed in patients undergoing general anesthesia. The remimazolam Ce significantly correlated with sedation depth. The Ce_50_ for MOAA/S scale ≤1 and ROR were determined to be 0.654 and 0.368 μg/mL, respectively.

## 1 Introduction

Remimazolam, a recently approved ultra-short-acting benzodiazepine, has been successfully used for general anesthesia and procedural sedation. This agent is rapidly metabolized into inactive metabolites by carboxylesterase, thereby presenting dose-dependent control over the depth and duration of sedation (1). Its pharmacokinetic attributes, including a limited steady-state volume of distribution, brief elimination half-life, and linear kinetics, contribute to its rapid and predictable onset and recovery from action (2, 3).

Pharmacokinetic and pharmacodynamic (PKPD) models of remimazolam have recently been constructed to allow target-controlled infusion (TCI) of effect-site concentration (Ce) (2, 4, 5). However, most of these models are based on young healthy volunteers, and predictive Ce for TCI has rarely been used in clinical settings. Thus, the quantitative relationship between remimazolam Ce and the depth of anesthesia in the clinical setting remains unclear. Therefore, exploring the dynamics of remimazolam Ce across various sedation levels and during recovery from general anesthesia could enhance the utility of remimazolam TCI in total intravenous anesthesia (TIVA) for surgical patients.

In clinical settings, the bispectral index (BIS) is commonly used to measure the depth of anesthesia in patients undergoing general anesthesia. However, the adequacy of the BIS in reflecting the sedation levels or depth of general anesthesia induced by remimazolam has not been fully established. Previous studies have shown that the BIS is not sensitive enough to accurately reflect the sedation levels in patients receiving midazolam, a benzodiazepine similar to remimazolam (6, 7).

Therefore, in this study, we aimed to investigate the relationship between remimazolam Ce and sedation levels or emergence from anesthesia in patients undergoing general anesthesia, using pharmacodynamic modeling. We also aimed to find the correlation between remimazolam Ce and sedation level or BIS, to determine the predictive probability (*P*_*K*_) of remimazolam Ce and BIS for detecting sedation levels, and to investigate the patient factor significantly correlated with remimazolam Ce at loss of consciousness and recovery from anesthesia. We hypothesized that adequate pharmacodynamic models between remimazolam Ce and Modified Observer’s Assessment of Alertness/Sedation scale (MOAA/S scale) or recovery from general anesthesia can be made in clinical setting.

## 2 Materials and methods

### 2.1 Study population and ethical approval

This single-arm, single-center, prospective clinical trial was approved by the Institutional Review Board of the Catholic University of Korea, Yeouido St. Mary’s Hospital (SC22OISI0205) and registered with the Clinical Trial Registry of the Korea National Institute of Health (CRIS,^1^ identification number: KCT0008210). Written informed consent was obtained from all participants prior to the study.

Patients aged 20–75 years with an American Society of Anesthesiologists (ASA) physical status of I or II scheduled for minimally invasive surgery (duration < 2 h) under general anesthesia were enrolled. The exclusion criteria included uncontrolled obstructive lung disease (forced expiratory volume in one second <50% of predicted or taking oral steroids), uncontrolled hypertension (resting blood pressure > 140/90 mmHg), chronic kidney disease ≥stage 3, hepatic dysfunction (serum aminotransferases >100 U/L), diagnosed cerebrovascular disease (stroke, cerebral aneurysm, cerebral hemorrhage, and carotid artery disease), medication affecting the central nervous system (opioids, anticonvulsants, anxiolytics, antiparkinson agents, sedatives, and hypnotics), history of allergic reactions to benzodiazepines or remifentanil, body mass index > 30 kg/m^2^, and pregnancy or nursing.

### 2.2 Study protocol

The patient did not receive any premedication. Upon arrival in the operating room, the electrocardiogram, peripheral oxygen saturation (SpO_2_), non-invasive arterial pressure, and BIS (BIS^®^ monitor, Covidien Medical, Boulder, CO, USA) were monitored and recorded throughout the procedure using vital sign recorder software (VitalDB,^2^ last accessed: August 2023).

Remimazolam (Byfavo™ Inj; Hana Pharm Co., Ltd, Hwaseong, South Korea) was prepared by dilution with 50 mL of 0.9% sodium chloride to achieve a concentration of 2 mg/mL (100 mg of remimazolam mixed with normal saline 50 mL). Preoxygenation was applied with 100% oxygen through a facial mask. Anesthesia was induced with remimazolam via a syringe pump (Pion pump, Bionet, Seoul, Korea) controlled by Asan pump software (version 2.1.5,^3^ last accessed August 2012) programmed with the PKPD model reported by Schüttler et al. (2). The blood-brain equilibration rate constant (k_e0_) for MOAA/S scale was used, whose value is 0.27 (min^–1^).

The sedation level was evaluated using the MOAA/S scale (0 = does not respond to deep stimuli; 1 = does not respond to mild prodding or shaking; 2 = responds only after mild prodding or shaking; 3 = responds only after the name is called loud or repeatedly; 4 = lethargic response to name spoken in normal tone; 5 = responds readily to name spoken in normal tone) (8). The remimazolam Ce predicted by the Asan pump software initially targeted 0.25 μg/mL and was sequentially increased by 0.05 μg/mL until the sedation level reached MOAA/S scale ≤1. Each time the target Ce was reached, the BIS was recorded and then the MOAA/S scale was checked. Then the target Ce was increased. Loss of consciousness (LOC) is defined as MOAAS ≤ 1.

After reaching a sedation level of MOAA/S scale 1, a continuous infusion of remifentanil (0.2–0.5 μg/kg/min) was initiated. The target Ce was then increased by 0.05–0.1 μg/mL from the Ce at MOAA/S scale 1, and rocuronium (0.6 mg/kg) was administered to facilitate endotracheal intubation. Following endotracheal intubation, the target Ce of remimazolam and the infusion rate of remifentanil were adjusted to maintain a BIS of 40–70 and a heart rate within 20% of the baseline value. When the mean arterial pressure was <60 mmHg or the systolic arterial pressure fell below 90 mmHg, ephedrine (5–10 mg) was administered intravenously. Conversely, if systolic arterial pressure exceeded 160 mmHg, nicardipine (0.5 mg) was administered intravenously. If the heart rate dropped below 45 beats/min, atropine (0.5 mg) was administered intravenously.

Approximately 5 min before surgery completion, the target Ce of remimazolam was reduced by approximately 20–30%. Subsequently, remimazolam and remifentanil infusions were discontinued at the end of surgery. Sugammadex (2 mg/kg) was administered to reverse the neuromuscular blockade.

The Ce of remimazolam was recorded when the patient responded to verbal stimuli and mild shaking (defined as recovery of responsiveness [ROR]) and at the time of endotracheal extubation (indicating full recovery of self-respiration). If the patient did not awaken within 15 min of remimazolam discontinuation, 0.2 mg of flumazenil was administered intravenously. Subsequently, patients were transferred to the post-anesthesia care unit for recovery.

The BIS was continuously monitored and recorded every 10 s to investigate the highest and lowest values during surgery as well as the differential between these two values for each patient.

### 2.3 Primary outcome

Using pharmacodynamic modeling, we aimed to determine the Ce of remimazolam at which each sedation level was reached with 50% probability, and the Ce at which ROR and endotracheal extubation were achievable with 50% probability upon awakening from anesthesia.

### 2.4 Secondary outcome

The secondary outcome was the correlation between sedation level and remimazolam Ce or BIS and the prediction probability of Ce or BIS for detecting sedation levels. We also aimed to investigate the correlation between patient factors and Ce at MOAA/S scale 1 and ROR.

### 2.5 Probability of sedation or emergence from anesthesia

The relationship between the Ce of remimazolam and the probability of a given sedation level was analyzed using the sigmoid Emax model. The model is defined as follows:


$$P\left(\text{MOAA}/\text{S}\leq \text{n}\right)=\frac{C\,e^{\gamma }}{C\,e^{\gamma }+C\,e_{50\left(MOAA/S\leq \text{n}\right)}^{\gamma }}$$

where P (MOAA/S ≤ n) represents the probability of a sedation level equal to or deeper than a given MOAA/S scale (n); Ce_50(MOAA/S__≤__n)_ is the steady-state Ce associated with a 50% probability for MOAA/S scale ≤ n; and γ represents the slope of steepness for probability versus the Ce curve. A pharmacodynamic model was developed for MOAA/S scales 1 to 4.

Using the observed ROR or endotracheal extubation, the response was categorized as 0 (no response or intubation status) or 1 (ROR or endotracheal extubation) in a pharmacodynamic model of emergence from general anesthesia. The relationship between the probability of ROR or extubation and remimazolam Ce was analyzed using a sigmoidal Emax model.


$$P\,\text{(ROR or extubation)}=1-\frac{C\,e^{\gamma }}{C\,e^{\gamma }+C\,e_{50\,R\,O\,R\,o\,r\,e\,x\,t\,u\,b\,a\,t\,i\,o\,n}^{\gamma }}$$

where P is the probability of ROR or endotracheal extubation, Ce_50_ is the Ce associated with 50% probability of ROR or extubation, and γ is the steepness of the concentration-versus-probability relationship.

The likelihood (L) of an observed response (R) can be described using the following equation:


Likelihood=R×P+(1-R)×(1-P)

where P represents the probability of sedation [P(MOAA/S scale ≤ n)], ROR (P(ROR)), or extubation (P(extubation)). Model parameters were estimated using the ‘LIKELIHOOD LAPLACE METHOD = conditional’ in the nonlinear mixed-effects modeling software (NONMEM^®^ 7 level 4, ICON Development Solutions, Dublin, Ireland). Interindividual random variability for Ce_50_ or gamma was estimated using a log-normal method or was fixed at zero. Internal validation was conducted with a non-parametric bootstrap procedure using Fit4NM [version 4.6.0 (see text footnote 3); last accessed June 2014], and the original dataset was randomly sampled to generate 2000 bootstrap replicates. The 95% confidence intervals of the nonparametric bootstrap replicates were obtained and compared with the final model parameter estimates. All model parameters were reported as typical values with relative standard errors (RSE) and 95% confidence intervals (CIs) derived from log-likelihood profiling.

### 2.6 Statistical analysis

To assess the ability of remimazolam Ce or BIS to predict the MOAA/S scale during anesthetic induction, *P*_*K*_ was calculated using Kim’s d cross-tabulation statistic (9). The pharmacokinetic tool program Fit4NM 4.6.0 (Eun-Kyung Lee and Gyu-Jeong Noh^4^ ; last accessed: October 2012) was utilized for this purpose. *P*_*K*_ was calculated as follows:


$$P_{K}=\left({\mathrm{Somers}}^{\prime }\text{d}+1\right)/2$$

A *P*_*K*_ value of 0.5 indicated that the parameter has no better than a 50:50 chance of correctly predicting an event, whereas a *P*_*K*_ value of 1.0 indicates 100% prediction accuracy.

Statistical analyses were performed using SPSS (version 24.0; SPSS Inc., Chicago, IL, USA) for Windows (Microsoft Corporation, Redmond, WA, USA). The correlation between the MOAA/S scale and BIS or remimazolam Ce was analyzed using linear regression and Pearson’s correlation analyses. Correlations between remimazolam Ce at LOC (defined as MOAAS ≤ 1) or ROR during emergence from anesthesia and several clinical variables were determined using Pearson’s correlation analyses. *P-*values < 0.05 were considered statistically significant.

## 3 Results

Sixty patients were screened, of which six were excluded from the study. Fifty-four patients met the inclusion criteria; however, four were dropped because of a connection error between the TCI program and syringe pump during anesthesia. Finally, 50 patients were enrolled in this study and effect-site TCI using Schüttler’s PKPD model was successfully implemented. The demographic and perioperative data of the enrolled patients are presented in Table 1. The surgeries that enrolled patients underwent included laparoscopic cholecystectomy (*n* = 30), hysteroscopy (*n* = 8), transurethral resection of bladder or prostate tumors (*n* = 7), and others (*n* = 5).

**TABLE 1:** Demographic and perioperative data of the patients.

Variables	Median (range)
Sex (male/female)	23/27
Age (years)	50 (22 – 75)
ASA physical status (I/II)	23/27
Body weight (kg)	64.53 (40.7 – 95.7)
Height (cm)	165.5 (150.7 – 182)
BMI (kg/m^2^)	23.13 (16.3 – 29.7)
Duration of operation (min)	32.5 (10 – 73)
Duration of anesthesia (min)	60 (40 – 95)
Remimazolam dose (mg)	55.96 (32.2 – 82.0)
Remimazolam infusion rate (mg/kg/h)	0.87 (0.51 – 1.74)
Remifentanil dose (μg)	580 (240 – 1140)
Remifentanil infusion rate (μg/kg/min)	0.15 (0.09 – 0.26)
Surgical distribution	
Laparoscopic cholecystectomy	30
Hysteroscopy	8
Transurethral resection of bladder or prostate tumors	7
Others	5

ASA: American Society of Anesthesiologists, BMI: body mass index, Remimazolam infusion rate: total dose administered during anesthesia (mg)/weight (kg)/anesthesia duration (hour), Remifentanil infusion rate: total dose administered during anesthesia (μg)/weight (kg)/anesthesia duration (min).

**FIGURE 1:**
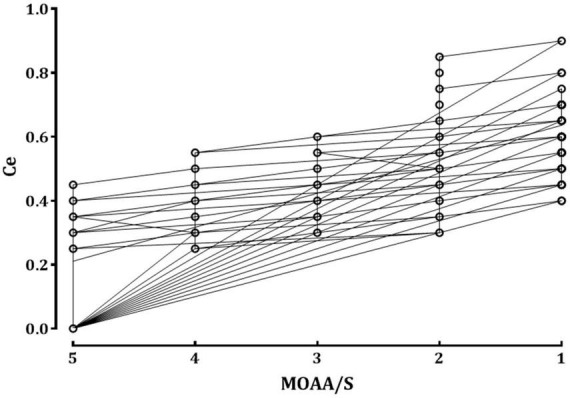
The relationship between the remimazolam effect-site concentration and sedation scale. The circles represent the raw data observed from all patients. Ce, effect-site concentration of remimazolam; MOAA/S: Modified Observer’s Assessment of Alertness/Sedation scale.

**FIGURE 2:**
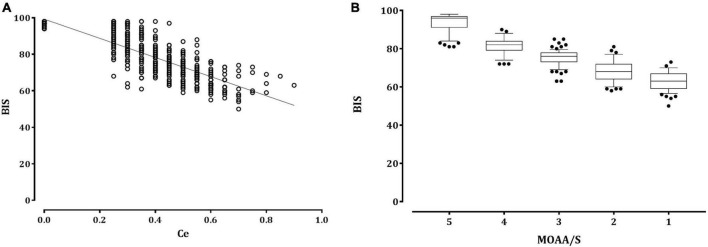
The relationship between the remimazolam effect-site concentration or sedation scale and the bispectral index. **(A)** Distribution of the bispectral index (empty circle) with the remimazolam effect-site concentration (Ce). The solid lines represent the linear regression of the bispectral index versus remimazolam Ce. **(B)** Box and whisker plots of BIS and Modified Observer’s Assessment of Alertness/Sedation scale scores. The upper and lower limits of the box indicate the 75th and 25th percentiles of the sample, respectively, and the horizontal line inside each box indicates the median. The upper and lower notches indicate the 90th and 10th percentiles of the samples, respectively. The values below and above the notches were drawn as individual points. BIS, bispectral index; Ce, effect-site concentration; MOAA/S, Modified Observer assessment of the alertness/sedation scale.

The relationship between the remimazolam Ce and the observed sedation levels is shown in Figure 1. An increase in remimazolam Ce was significantly correlated with an increase in the sedation levels, as depicted by the regression formula *Y* = −0.106 × X + 0.742 (correlation coefficient [*r*] = −0.793, *P* < 0.001). Figure 2 shows a significant negative correlation between the BIS value and the remimazolam Ce (*Y* = −52.46 × X + 99.23, *P* < 0.001; *r* = −0.8168, *P* < 0.001). The BIS was significantly correlated with the sedation levels (*Y* = 7.87 × X + 52.7, *P* < 0.001; *r* = 0.914, *P* < 0.001). The median BIS (range) at MOAA/S scales 5, 4, 3, 2, and 1 were 96 (81–98), 82 (72–90), 76 (63–85), 68 (58–81), and 63 (50–73), respectively (Figure 2B).

The *P*_*K*_ values, with 95% confidence intervals (CI) and standard errors (SE), for remimazolam Ce and BIS in detecting changes on the MOAA/S scale were 0.797 (95% CI, 0.783–0.810; SE, 0.007) and 0.756 (95% CI, 0.738–0.775; SE, 0.009), respectively. The *P*_*K*_ value for detecting BIS changes using the remimazolam Ce was 0.846 (95% CI, 0.827–0.864; SE, 0.010).

**FIGURE 3:**
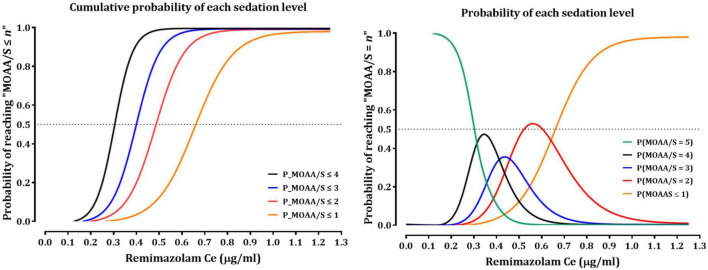
Probability of reaching each MOAA/S scale being equal to less than a given level (MOAA/S ≤ n) and each MOAA/S scale being equal to a given level (MOAA/S = n) as a function of predicted remimazolam effect site concentration based on the final pharmacodynamic model. MOAA/S, Modified Observer’s Alertness/Sedation scale; Ce, effect-site concentration.

**FIGURE 4:**
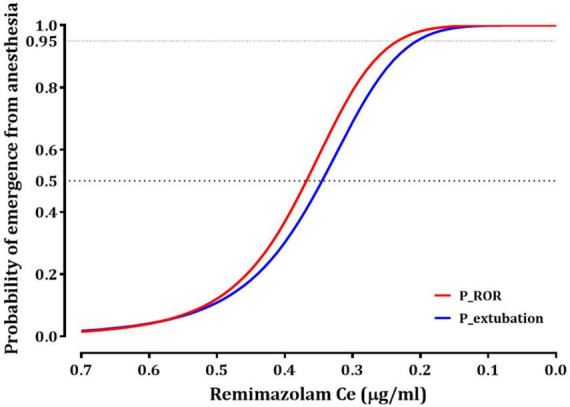
The relationship between the probability of recovery of responsiveness (ROR) or endotracheal extubation and effect-site concentration of remimazolam. Ce_50ROR_ and Ce_95ROR_ (effect-stie concentration of remimazolam, associated with 50 and 95% probability for ROR, respectively) are 0.368 and 0.232 μg/ml; Ce_50extubation_ and Ce_95extubation_ (effect-stie concentration of remimazolam, associated with 50 and 95% probability for extubation, respectively) are 0.345 and 0.204 μg/ml.

Tables 2, 3 summarize the pharmacodynamic parameter estimates and median parameter values (2.5–97.5% percentile) from the non-parametric bootstrap replicates of the final pharmacodynamic model for each sedation level (MOAA/S scale) and emergence from anesthesia, respectively. The predicted remimazolam Ces associated with a 50% probability (Ce_50_) of LOC, ROR, and endotracheal extubation were 0.656, 0.368, and 0.345 μg/mL, respectively. Figure 3 depicts the probability of reaching each MOAA/S scale equal to or less than a given level (MOAA/S ≤ n) and each MOAA/S scale equal to a given level (MOAA/S scale = n) as a function of the predicted remimazolam Ce, based on the final pharmacodynamic model. Figure 4 shows the pharmacodynamic relationship among the probability of ROR, extubation, and remimazolam Ce. The remimazolam Ces associated with a 95% probability for ROR and endotracheal extubation (Ce_95ROR_ and Ce_95extubation_) were 0.232 and 0.204 μg/ml, respectively. The median BIS scores at ROR and extubation (range) were 78 (71–91) and 80.5 (73–92), respectively.

**TABLE 2:** Estimates of pharmacodynamic parameters and median parameter values (2.5–97.5%) of the non-parametric bootstrap replicates of the pharmacodynamic model for each level of sedation (MOAA/S scale).

Parameter	Estimate (%RSE)	Median (2.5 – 97.5%) of bootstrap replicates
Ce_50MOAAS ≤ 4_	0.302 (3.510)	0.305 (0.29 – 0.318)
Ce_50MOAAS ≤ 3_	0.397 (3.123)	0.402 (0.386 – 0.417)
Ce_50MOAAS ≤ 2_	0.483 (2.629)	0.487 (0.471 – 0.503)
Ce_50MOAAS ≤ 1_	0.654 (3.792)	0.664 (0.634 – 0.692)
γ	7.74 (10.271)	7.72 (6.840– 8.92)

No interindividual random variability was assumed. The non-parametric bootstrap analysis was repeated 2000×. MOAA/S scale: Modified Observer’s Alertness/Sedation scale; MOAA/S ≤ n: MOAA/S score being equal to or less than a given level ‘n’; Ce_50MOAA/S ≤ n_: effect-site concentration of remimazolam (μg/mL) associated with 50% probability of ‘MOAA/S ≤ n’; γ: the slope steepness for the relationship of the effect-site concentration vs. ‘MOAA/S ≤ n’; RES, relative standard error (SE) = SE/mean × 100 (%).

**TABLE 3:** Estimates of pharmacodynamic parameters and median parameter values (2.5–97.5%) of the non-parametric bootstrap replicates of the pharmacodynamic model for remimazolam anesthesia at emergence from anesthesia.

Parameter	Estimate (%RSE)	Median (2.5 – 97.5%) of bootstrap replicates
Ce_50ROR_	0.368 (3.913)	0.372 (0.353 – 0.39)
γ	5.67 (12.998)	5.74 (4.89 – 6.930)
Ce_50extubation_	0.345 (3.623)	0.349 (0.332 – 0.364)
γ	6.47 (12.241)	6.43 (5.55 – 7.62)

No interindividual random variability was assumed. The non-parametric bootstrap analysis was repeated 2000×. Ce_50ROR_: effect-site concentration of remimazolam associated with a 50% probability of the return of response to stimulation. Ce_50extubation_: effect-site concentration of remimazolam associated with 50% probability of endotracheal extubation.

Table 4 shows correlation coefficients between several clinical variables and remimazolam Ce at LOC or ROR during emergence from anesthesia. The remimazolam Ce at LOC was significantly correlated with age (Linear regression: *Y* = −90.8 × X + 106, *P* < 0.001), and the remimazolam Ce at ROR was significantly correlated with age (Linear regression: *Y* = −113 × X + 92.1, *P* < 0.001) and total remimazolam dose administered (Linear regression: *Y* = 71.6 × X + 30.3, *P* < 0.001).

**TABLE 4:** Correlation coefficients between several clinical variables and remimazolam effect-site concentration at loss of consciousness (Modified Observer’s Alertness/Sedation scale 1) during induction or return of responsiveness during emergence from anesthesia.

Variables	Correlation coefficient with Ce at LOC	*P*-value	Correlation coefficient with Ce at ROR	*P*-value
Age (years)	-0.60	<0.001	-0.643	<0.001
Body weight (kg)	0.01	0.935	0.075	0.603
Height (cm)	0.17	0.238	0.228	0.112
BMI (kg/m^2^)	-0.102	0.483	-0.061	0.674
Duration of operation (min)			0.028	0.849
Duration of anesthesia (min)			-0.087	0.547
Remimazolam dose (mg)			0.562	<0.001
Remifentanil dose (μg)			0.184	0.202

LOC, loss of consciousness; ROR, return of responsiveness.

Variability was observed in the changes in BIS during surgery. The median values (range) for the nadir and highest BIS were 41.65 (28.2–57) and 67.6 (56.9–78.9), respectively. The median difference between the nadir and highest BIS was 25.55 (12–44.3).

Two patients received a single intravenous dose of flumazenil (0.2 mg). Hypertension, hypotension, and bradycardia occurred in 8, 2, and 1 patients, respectively. When questioned in the recovery room, none of the patients reported awareness or memory of the anesthesia.

## 4 Discussion

In this study, we explored the pharmacodynamic relationship between the Ce of remimazolam and the sedation levels, as well as emergence from general anesthesia, in patients receiving remimazolam through TCI for TIVA. Our findings indicate that the predicted Ce for LOC and ROR were 0.654 and 0.368 μg/mL, respectively. Remimazolam Ce and BIS significantly correlated with the sedation levels and comparably predicted the sedation level, whereas BIS fluctuated during surgery. The findings of this study can be helpful for the adjustment of target Ce during procedural sedation or TIVA.

A comparison of traditional zero-order infusion with TCI underscores its superior precision and control in administering anesthetic drugs. This methodology facilitates individualized drug administration, considering patient-specific factors, such as weight, age, and laboratory findings, thereby minimizing the risks associated with over- or under-dosing (10). The TCI maintains steady drug concentrations, enabling clinicians to objectively evaluate changes in concentration-dependent effects and more accurately adjust drug dosages across the entire therapeutic range. The linear pharmacokinetics of remimazolam, characterized by high clearance, small steady-state volume of distribution, and a brief terminal half-life, render it particularly suitable for TCI (2, 3). Especially clearance is known to be independent of body weight and have small interindividual variability (1). Schüttler et al. developed a three-compartment PK model in which body weight was included as a covariate, which significantly affected the volume of distribution in the central compartment (2). Their model can be applied for remimazolam TCI, but there are few studies in the clinical setting. Nevertheless, for effective application of TCI in clinical settings, it is imperative to ascertain the therapeutic ranges of Ce that correspond to the targeted sedation levels or general anesthesia.

We assessed the Ce_50_ for each level of sedation using the MOAA/S scale, a widely used clinical tool for sedation assessment. The MOAA/S scale categorizes sedation levels as minimal (score 4), moderate (score 3), deep (scores 2–1), and general anesthesia (score 0). Shuttler et al. reported that the Ce_50_ at MOAA/S scale 0 was more than double that at MOAA/S scale 1 (2); and assessing MOAA/S scale 0 in clinical patients is challenging without the use of analgesics because application of pain stimulation is difficult. In clinical practice, remimazolam for TIVA is often co-administered with remifentanil, an opioid that exhibits significant synergism with remimazolam (11). Therefore, we titrated the Ce only to MOAA/S scale 1 and initiated remifentanil administration for endotracheal intubation.

Schüttler et al. estimated the Ce_50_ for MOAA/S scale of 4, 3, 2, and 1 in young, healthy male volunteers to be 0.337, 0.481, 0.506, and 0.64 μg/ml, respectively (2). Our findings indicated similar Ce_50_ values during sedation but slightly lower values at MOAA/S scale 4, 3, and 2. This discrepancy might be attributed to the older age of our study participants compared with those included by Shuttler et al., potentially leading to a lower Ce_50_ for mild and moderate sedation levels. However, similar to the results of Schüttler et al., there was a lot of overlap in Ce50 values for MOAA/S scale ≤ 4, 3, and 2 (Table 2). The Hill coefficient (γ) of the concentration-response curve was 7.74 in this study, which is steeper than the findings of Schüttler et al. (3.6), suggesting that unwanted deep sedation may occur more easily in the clinical setting (2). Although it may be difficult to titrate Ce values for moderate to deep sedation when applying TCI to sedated patients, the results of this study can be used as reference values. A simulation study by Kim et al. explored Ce at loss of responsiveness (LOR), defined as unresponsiveness to loud verbal commands, using Shuttler’s model. The median simulated Ce of remimazolam at LOR was 0.5 and 0.8 μg/ml for patients receiving dosages of 6 mg/kg/h and 12 mg/kg/h, respectively (12). These values were higher than our observed Ce_50_ values for MOAA/S scale 2. Notably, patients receiving remimazolam at 12 mg/kg/h achieved LOR significantly faster than those receiving remimazolam at 6 mg/kg/h (12). This observation supports the notion that fixed-dose remimazolam regimens can lead to considerable variability in clinical sedation outcomes.

The relationship between remimazolam administration and respiratory function during and after general anesthesia is a critical aspect of anesthesia management. When used for sedation or induction of general anesthesia, remimazolam is associated with respiratory depression following LOC in most patients (13). In contrast, during emergence from remimazolam-based TIVA, a distinct pattern is observed in which spontaneous breathing may not fully recover even after the return of consciousness. This phenomenon has been consistently reported in previous studies, where recovery of consciousness, typically marked by eye opening, generally precedes endotracheal extubation by approximately 0.4–1 min (14, 15). In our study, we found that the Ce_50ROR_ was 0.368 μg/mL, which was higher than the Ce_50extubation_ (0.345 μg/mL). This finding suggests that most patients in our study responded to stimuli prior to the full restoration of respiratory function during emergence from anesthesia. Furthermore, the effective concentrations for 95% of the patients (Ce_95_) for ROR and extubation were 0.232 and 0.204 μg/mL, respectively. These values were approximately 0.1 μg/mL lower than the Ce_50_ for a MOAA/S sccale of ≤4, indicating a more conservative threshold for ensuring patient safety during recovery from anesthesia. Considering these observations, it is evident that careful adjustment and monitoring of the predicted Ce for remimazolam TCI are essential for safe recovery from remimazolam TIVA. These adjustments should be informed by an understanding of the relationship among remimazolam concentration, consciousness, and respiratory function during emergence from anesthesia.

Since determining sedation levels by sedation scales requires stimulation that may interfere with the procedure, noninvasive electroencephalography-guided monitoring may help assess the level of sedation. In this study, a significant correlation was observed between the level of sedation and BIS during anesthesia induction, with a Pearson’s correlation coefficient (*r*) of 0.914. This finding aligns with that of a previous study by Zao et al., which also found a significant correlation between the MOAA/S scale and BIS following a single-bolus injection of remimazolam tosylate (16). However, both their data and those of our study showed considerable overlap in BIS values across different sedation levels. BIS is reportedly less predictive of sedation depth with midazolam than with propofol (6). Ibrahim et al. reported a *P*_*K*_ value of 0.69 for midazolam, which is notably lower than the *P*_*K*_ value of 0.87 for propofol sedation (6). In our study, the *P*_*K*_ value of BIS for the sedation level was 0.756, which was higher than the *P*_*K*_ value reported for midazolam, but lower than the *P*_*K*_ value for remimazolam Ce (0.797). Additionally, Eisenreid et al. reported that the *P*_*K*_ of the Narcotrend Index for the MOAA/S scale was 0.74, which was lower than the beta ratio (0.79) (17). These results suggest that indices used for monitoring the depth of anesthesia, such as the BIS, may not fully account for the distinct electroencephalographic characteristics of different anesthetics. Therefore, considering both the Ce of remimazolam and changes in values from depth of anesthesia monitors at each stage of sedation is advisable for a more comprehensive assessment of anesthetic depth.

Intraoperative BIS values tend to be relatively high during remimazolam TIVA (18, 19). Accordingly, in our study, we aimed for an intraoperative BIS target range of 40–70, which was higher than the typically recommended range for general anesthesia. Despite this targeted range, we noticed considerable variability in both the peak BIS and BIS ranges among individual patients during surgical procedures. In support of this observation, a phase IIb/III trial revealed that the mean intraoperative BIS ranged from 40 to 84 during remimazolam TIVA (18). Notably, their study reported no signs of intraoperative awakening or postoperative recall, despite elevated BIS values (18). Similarly, a study on a Korean cohort (*n* = 1500) found that in approximately 4.1% of patients, the BIS did not fall below 60, even when remimazolam was administered at the maximum recommended dose for the induction or maintenance of anesthesia (20). These findings underscore the need for further research in diverse clinical settings to determine the optimal BIS range for remimazolam-induced general anesthesia.

In this study, we fixed inter-individual variability for Ce_50MOAA/S__≤__n_ and γ as zero to avoid over-parameterization. However, when modeling emergence from anesthesia and attempting to incorporate the interindividual variability of Ce_50ROR_ or Ce_50extubation_, the covariance step could not be processed. Minimization of the objective function value (OFV) was successful only when estimating the inter-individual variability for γ. Despite attempts to include age as a covariate for γ, the OFV did not decrease significantly. Consequently, we adopted a naïve pooled data approach for all final models, disregarding individual data correlations.

Nonetheless, we explored the relationship between clinical variables and remimazolam Ce at the LOC and ROR. Our findings indicated that age was inversely correlated with remimazolam Ce at both the LOC and ROR. Prior pharmacokinetic studies have shown that factors such as adjusted body weight, sex, and ASA physical status can significantly influence elimination clearance (11, 21). The effect of age on the pharmacokinetics of remimazolam remains unclear. Zhou et al. found no significant effect of age on remimazolam clearance, whereas Masui et al. identified age as a significant covariate affecting pharmacokinetic parameters. However, pharmacodynamic studies have demonstrated the influence of age on the time to LOC and induction dose (13, 22). Chae et al. reported that older age was significantly associated with lower 50% effective dose for LOC and respiratory depression onset (13). Pharmacodynamic studies have shown that a 75-year-old patient may have LOC onset 5–10 s sooner than a 30-year-old patient following a dose of remimazolam 6 mg/kg/h (22). A previous simulation study also highlighted a significant relationship between age and simulated Ce at ROR, suggesting that elderly patients may recover with a lower remimazolam Ce based on population pharmacodynamic modeling (12). Therefore, age is an important factor to consider when administering remimazolam. Further studies may be needed to determine the mathematical relationship between the target Ce during sedation or general anesthesia and the patient’s age.

In our study, the total infused remimazolam dose was positively correlated with remimazolam Ce at ROR, meaning that ROR was achieved at higher Ce in patients who received higher remimazolam doses. This result is thought to be influenced by confounding factors such as the patient’s age. Previous studies have reported that the time to awakening/extubation after remimazolam TIVA was not affected by the surgery duration or total remimazolam dose; thus, remimazolam had no cumulative sedative effects (11, 22). Our study, unlike previous studies, focused only on patients who underwent brief surgical procedures. Given these limitations, this finding may not be generalizable to situations involving longer surgeries. Therefore, further research is needed in the context of longer surgical procedures to clarify the effect of surgical duration and total remimazolam dose on the recovery phase.

This study had several limitations. First, the accuracy of Shuttler’s model in predicting the effect-site concentration (Ce) of remimazolam was assumed. Therefore, the actual variability in Ce values corresponding to different sedation depths may be greater than that reported in this study. This reliance on a single model for Ce prediction could limit the applicability of our findings to a broader clinical context. Second, the pharmacodynamic (PD) modeling conducted in this study did not include a search for covariates. The incorporation of covariates into the PD model may have provided a more nuanced mathematical representation of the relationship between patient-specific factors and the Ce of remimazolam during TCI. The exclusion of covariates may have limited our ability to fully understand and predict the pharmacodynamic behavior of remimazolam in a patient-specific manner. Third, there was variability in the types and progress of surgeries among the patients included in the study. This heterogeneity may influence the pharmacodynamics of remimazolam, particularly during recovery from anesthesia. Additionally, the concomitant use of remifentanil and its varying effects can further complicate the recovery process. The extent of postoperative pain, which varies depending on the type of surgery, may also have affected the Ce of remimazolam during the ROR. Thus, the influence of surgical factors and adjunctive analgesic use on remimazolam pharmacodynamics were not fully addressed in the present study.

## 5 Conclusion

In conclusion, this study presented Ce_50_ for attaining MOAA/S scale ≤ 4, 3, 2 and 1, and recovery from general anesthesia in patients undergoing remimazolam TCI. The Ce_50_ for MOAA/S scale ≤ 1 and ROR were determined to be 0.654 and 0.368 μg/mL, respectively. We found a strong correlation between remimazolam Ce and sedation level, suggesting that its combined use with BIS is a reliable guide for anesthesia administration. However, further research is required to validate this approach in different patient populations and clinical settings.

## Data availability statement

The raw data supporting the conclusions of this article will be made available by the authors, without undue reservation.

## Ethics statement

The studies involving humans were approved by the Institutional Review Board of the Catholic University of Korea, Yeouido St. Mary’s Hospital. The studies were conducted in accordance with the local legislation and institutional requirements. The participants provided their written informed consent to participate in this study.

## Author contributions

JC: Project administration, Funding acquisition, Writing–review and editing. KS: Conceptualization, Data curation, Formal analysis, Methodology, Writing–original draft. JL: Data curation, Investigation, Writing–review and editing. SL: Data curation, Writing–review and editing.

## Footnotes

1
http://cris.nih.go.kr

2
https://vitaldb.net

3
https://www.ksap.co.kr/html/

4
https://cran.r-project.org/src/contrib/Archive/fit4NM/
